# Utility of blood biomarkers in inpatients with West Nile virus infection: an exploratory study

**DOI:** 10.3389/fmed.2026.1783007

**Published:** 2026-03-12

**Authors:** Giuseppe Cardillo, Carmine Siniscalchi, Maria Gabriella Coppola, Egidio Imbalzano, Rodolfo Nasti, Pierpaolo Di Micco

**Affiliations:** 1 Laboratory Unit, MedyLab srl, Bologna, Italy; 2 Department of Internal Medicine, University Hospital, Parma, Italy; 3 Internal Medicine Unit, Benevento, Italy; 4 Department of Clinical and Experimental Medicine, University of Messina, Messina, Italy; 5 Internal Medicine Unit, Ospedale Evangelico Villa Betania, Napoli, Italy; 6 UOC Medicina Interna, Pozzuoli, NA, Italy

**Keywords:** inflammatory indices, lymphocytopenia, neuroinvasive infection, routine laboratory biomarkers, sepsis, West Nile virus

## Abstract

**Background:**

West Nile virus (WNV) infection is an emerging public health concern in Southern Europe and may present with heterogeneous clinical phenotypes, ranging from predominantly neurologic manifestations to systemic inflammatory and septic presentations. While lymphocytopenia and immune dysregulation are well-recognized features of WNV infection, data on routine hematological parameters and composite inflammatory indices, and their potential association with different clinical presentations, remain limited.

**Methods:**

We retrospectively analyzed a multicenter Italian cohort of 30 consecutive inpatients with confirmed neuroinvasive WNV infection. Patients were classified according to their predominant clinical presentation at admission as neurologic or septic phenotype. Baseline clinical characteristics, routine hematological parameters, routine hemostasis tests and composite inflammatory indices were collected. Between-group comparisons were performed using non-parametric tests. Univariable logistic regression was used to screen for laboratory variables associated with the septic phenotype, and a parsimonious multivariable model including only significant predictors was then fitted.

**Results:**

Seventeen patients (56.7%) presented with a predominantly neurologic phenotype and 13 (43.3%) with a septic phenotype. Median age was 77 years, and baseline comorbidities were broadly similar between groups. Both phenotypes showed marked systemic inflammation, lymphocytopenia and elevated composite inflammatory indices. Patients with a septic presentation displayed numerically higher neutrophil-to-lymphocyte ratio, systemic immune-inflammation index and platelet-related inflammatory indices, although these differences did not reach statistical significance. In univariable logistic regression analyses, lower mean corpuscular hemoglobin (MCH) and lower monocyte percentage were significantly associated with the septic phenotype. In a parsimonious multivariable model, lower MCH remained independently associated with septic presentation (OR 0.41, 95% CI 0.18–0.95), whereas monocyte percentage showed a non-significant trend.

**Conclusion:**

In this exploratory cohort of patients with neuroinvasive WNV infection, subtle alterations in erythrocyte indices and monocyte counts were associated with a septic clinical presentation, whereas inflammatory indices showed only non-significant trends. These findings suggest that routine hematological parameters may contribute to the early phenotypic characterization of WNV infection. Larger, multicenter studies are warranted to confirm these observations and to clarify the prognostic value of routine hematological and inflammation-related biomarkers in this setting.

## Background

West Nile fever (WNF) is an infection caused by a flavivirus that primarily circulates among birds and is transmitted to humans through the bite of infected mosquitoes. Owing to the ecological interaction between avian reservoirs and mosquito vectors, WNF is typically considered a seasonal infectious disease, with peak incidence during warmer months (1, 2). In Europe, WNV activity has intensified over the last decade, with marked seasonal outbreaks and geographic expansion, including the unprecedented 2018 transmission season characterized by an unusually early start and a steep increase in notified cases. These trends are consistent with the increasing suitability of European ecosystems for mosquito-borne infections and reinforce the clinical relevance of recognizing heterogeneous hospital presentations (3, 4). Recent reviews focusing on Southern Europe have further highlighted the sustained circulation of WNV and the importance of integrating clinical suspicion with laboratory patterns in hospitalized patients, particularly older and frail individuals (5). Nevertheless, sporadic cases may occur outside the usual transmission season, particularly in individuals with a history of recent travel to endemic areas (6). As humans are incidental and dead-end hosts, the clinical spectrum of West Nile virus (WNV) infection is highly heterogeneous, ranging from asymptomatic seroconversion to severe neuroinvasive disease, including encephalitis and encephalomyelitis with poor prognosis (7, 8). In addition to vector-borne transmission, iatrogenic infections have been reported through blood transfusion, solid organ transplantation and laboratory exposure, as well as vertical transmission via placenta or breast milk (9).

Symptomatic patients frequently present with neurological manifestations, often accompanied by diffuse myalgias and musculoskeletal pain, which may mimic other infectious or inflammatory conditions (10). Clinically overt disease is more commonly observed in frail individuals, particularly older adults and patients with significant comorbidities, including cardiovascular and immunological disorders (8, 11). Conversely, oligosymptomatic or asymptomatic patients may experience non-specific symptoms such as fever, headache, back pain, myalgia and anorexia lasting several days, making early clinical recognition challenging (2, 7).

Similar to other viral infections, WNV may induce lymphocytopenia and broader immune dysregulation (12). Potentially predisposing patients to secondary bacterial or fungal infections. In this context, some patients, especially those with an initially mild or oligosymptomatic presentation, may subsequently develop signs and symptoms consistent with sepsis rather than the classic cutaneous rash associated with arboviral infections (11, 13).

In the typical clinical course, following an incubation period ranging from 2 to 15 days, neurological signs and symptoms may emerge as a result of direct viral injury to the central nervous system. Meningoencephalitis is more frequently observed than isolated meningitis and may be associated with acute flaccid paralysis due to involvement of anterior horn cells of the spinal cord (7, 8). Cerebrospinal fluid analysis remains the cornerstone of diagnosis, allowing detection of viral RNA by polymerase chain reaction, while serological testing for anti-WNV IgM antibodies and magnetic resonance imaging of the brain and spinal cord provide valuable complementary diagnostic information (6, 14).

The aim of the present exploratory study was to evaluate whether routinely available hematological and inflammatory parameters at hospital admission are associated with distinct clinical phenotypes (neurologic versus septic) in patients with neuroinvasive WNV infection.

## Materials and methods

We retrospectively analyzed the medical records of 30 patients admitted to internal medicine wards of four different hospitals in Southern Italy for fever of unknown origin. Patients were identified at the time of admission to the participating hospitals and subsequently followed during their stay in internal medicine wards. All patients showed serological positivity for anti-West Nile virus IgM and IgG antibodies; molecular testing was also performed and confirmed viral infection. In 13 patients, the clinical course after admission was characterized by a presentation resembling a septic illness, with fever, cough, tachypnoea and urinary symptoms. In the remaining 17 patients, the clinical picture was predominantly neurologic, with manifestations including altered mental status, nausea and vomiting, headache, opisthotonus and axial rigidity. The baseline clinical characteristics of the study cohort are reported in Table 1. Blood samples collected at admission were analyzed to assess routine hematological parameters, composite inflammatory indices and routine hemostatic variables. For all patients with neurological dysfunction or clinically overt encephalitis, neuroimaging was also performed to confirm the diagnosis.

**Table 1:** Baseline characteristics of patients with neuroinvasive West Nile virus infection according to clinical phenotype.

Variable	Overall (*n* = 30)	Neurologic phenotype (*n* = 17)	Septic phenotype (*n* = 13)	*p* value
Age, years	77.00 [66.50–81.00]	73.00 [64.00–80.00]	78.00 [72.00–84.00]	0.367
Male sex	16/30 (53.3%)	8/17 (47.1%)	8/13 (61.5%)	0.484
COPD	7/30 (23.3%)	2/17 (11.8%)	5/13 (38.5%)	0.190
Heart failure	19/30 (63.3%)	12/17 (70.6%)	7/13 (53.8%)	0.454
Chronic dialysis	2/30 (6.7%)	1/17 (5.9%)	1/13 (7.7%)	1.000
Active cancer	7/30 (23.3%)	5/17 (29.4%)	2/13 (15.4%)	0.427
Baseline thrombocytopenia	3/30 (10.0%)	1/17 (5.9%)	2/13 (15.4%)	0.565
Platelets, ×10^3^/μL	179.00 [125.50–218.25]	152.00 [124.00–193.00]	180.00 [140.00–429.00]	0.225
Normal PT	24/30 (80.0%)	13/17 (76.5%)	11/13 (84.6%)	0.672
Normal APTT	26/28 (92.9%)	15/17 (88.2%)	11/11 (100.0%)	0.505
Antithrombotic therapy	14/28 (50.0%)	7/17 (41.2%)	7/11 (63.6%)	0.440
Antiplatelet therapy	12/28 (42.9%)	5/17 (29.4%)	7/11 (63.6%)	0.121
Anticoagulant therapy	8/28 (28.6%)	6/17 (35.3%)	2/11 (18.2%)	0.419
Satellite lymphangitis	6/30 (20.0%)	4/17 (23.5%)	2/13 (15.4%)	0.672
Red blood cells, ×10^6^/μL	3.85 [3.65–4.35]	3.83 [3.21–4.38]	3.87 [3.71–4.27]	1.000
Hemoglobin, g/dL	11.30 [10.07–12.97]	11.40 [9.90–13.20]	11.20 [10.50–12.50]	0.897
Hematocrit, %	36.30 [32.15–40.15]	35.00 [31.80–40.50]	38.00 [33.30–39.50]	1.000
MCV, fL	83.75 [77.32–88.88]	85.10 [82.54–92.57]	80.00 [77.05–83.00]	0.077
MCH, pg	29.35 [28.00–30.45]	30.30 [29.35–31.00]	28.00 [26.25–28.61]	0.001
MCHC, g/dL	32.45 [30.96–33.80]	32.90 [31.36–33.83]	31.43 [30.05–33.11]	0.392
White blood cells, ×10^3^/μL	7.75 [6.42–10.12]	7.80 [5.87–9.90]	7.70 [6.90–11.20]	0.802
Neutrophils, cells ×10^3^/μL	3.38 [3.31–3.47]	3.35 [3.23–3.46]	3.40 [3.35–3.70]	0.146
Neutrophils, %	78.70 [77.00–81.50]	77.85 [75.08–80.30]	79.00 [78.00–85.00]	0.146
Lymphocytes, cells ×10^3^/μL	0.47 [0.34–0.55]	0.52 [0.43–0.58]	0.34 [0.26–0.69]	0.188
Lymphocytes, %	11.00 [8.00–13.60]	12.10 [10.25–13.40]	8.00 [6.50–14.00]	0.188
Monocytes, cells ×10^3^/μL	0.19 [0.16–0.33]	0.32 [0.19–0.43]	0.16 [0.086–0.17]	0.015
Monocytes, %	4.50 [3.00–7.60]	7.35 [4.55–9.50]	3.80 [2.50–4.00]	0.015
NLR	7.00 [6.12–10.25]	6.50 [5.31–7.50]	10.75 [4.88–13.07]	0.163
MLR	0.50 [0.38–0.67]	0.60 [0.37–1.00]	0.43 [0.17–0.57]	0.179
PLTLNFR	348.84 [225.29–441.86]	322.67 [225.29–420.11]	523.26 [180.88–2,166.1]	0.396
Positive anti IgM WNV	30/30 (100%)	17/17 (100%)	13/13 (100%)	0.500
Positive IgG anti WNV	30/30 (100%)	17/17 (100%)	13/13 (100%)	0.500
SII	1,211.29 [778.88–1,463]	1,128.39 [778.88–1,421.7]	1,935.00 [614.44–8,289.7]	0.396
SIRI	1.74 [1.27–2.25]	2.05 [1.27–3.53]	1.64 [0.57–2.06]	0.262
AISI	353.98 [157.41–447.25]	364.73 [157.41–464.6]	315.28 [99.50–499.49]	0.805

Neurologic phenotype: Septic = 0; septic phenotype: Septic = 1. COPD, chronic obstructive pulmonary disease; PT, prothrombin time; APTT, activated partial thromboplastin time; MCV, mean corpuscular volume; MCH, mean corpuscular hemoglobin; MCHC, mean corpuscular hemoglobin concentration; NLR, neutrophil-to-lymphocyte ratio; MLR, monocyte-to-lymphocyte ratio; PLTLNFR, platelet-to-lymphocyte-neutrophil fraction ratio; SII, systemic immune-inflammation index; SIRI, systemic inflammation response index; AISI, aggregate index of systemic inflammation.

Data are expressed as median [interquartile range] for continuous variables and *n*/*N* (%) for categorical variables.

*p* values were considered statistical significant if < 0.05.

Classification into septic versus neurologic phenotype was performed retrospectively based on review of the complete clinical records. Sepsis was defined according to Sepsis-3 criteria, as suspected or documented infection associated with an acute increase of ≥2 points in the Sequential Organ Failure Assessment (SOFA) score. Neuroinvasive disease was defined according to established clinical and laboratory criteria, including altered mental status and/or focal neurologic deficits with supportive cerebrospinal fluid and/or neuroimaging findings. Phenotype adjudication was performed by two independent clinicians at each center who reviewed the complete clinical record, including admission notes, laboratory data, microbiological results, imaging findings, and SOFA score components. In case of disagreement, classification was resolved by consensus discussion. No formal inter-observer agreement statistics were calculated due to the retrospective design and limited sample size.

For the purpose of the present analysis, only laboratory parameters measured at hospital admission were considered. Although repeated measurements were available for some patients during hospitalization, no longitudinal or time-dependent analyses were performed due to the limited sample size and heterogeneity in sampling intervals.

Hematological parameters were measured using automated hematology analyzers routinely employed at each participating center. Coagulation tests (prothrombin time [PT] and activated partial thromboplastin time [APTT]) and clinical chemistry parameters, including C-reactive protein, were performed using automated platforms according to local standard operating procedures. Due to the retrospective design and incomplete documentation of specific analyser models across centers, detailed instrument information could not be systematically reported. Although analyses were performed according to local standard operating procedures, minor inter-laboratory variability in measurement platforms and reference ranges cannot be excluded. No central laboratory re-analysis was performed.

## Statistical methodology

No formal sample size calculation was performed prior to study initiation. Given the exploratory and retrospective nature of the study, a convenience sample including all consecutive eligible patients during the study period was analyzed. All statistical analyses were performed using Python version 3.11 (pandas, scikit-learn, stats models and SciPy libraries). Data were assessed for plausibility and completeness. Variables with more than 50% missing data were excluded *a priori*, and all subsequent analyses were conducted using a complete-case approach. Binary variables were coded as 0 (absent) and 1 (present). Continuous variables are reported as median with interquartile range (IQR), whereas categorical variables are presented as counts and percentages.

Between-group comparisons were performed between patients with a predominantly neurologic phenotype (Septic = 0) and those with a septic phenotype (Septic = 1). The Mann–Whitney *U* test was used for continuous variables and Fisher’s exact test for categorical variables, in consideration of the small sample size. All statistical tests were two-tailed, and a *p* value < 0.05 was considered statistically significant. No adjustment for multiple comparisons was applied, given the exploratory nature of the study.

In multivariable analyses, monocyte percentage and absolute monocyte count were not entered simultaneously because the absolute count is derived from total WBC count and monocyte percentage, resulting in structural collinearity. Composite inflammatory indices were calculated as follows:

- The neutrophil-to-lymphocyte ratio (NLR);
- The monocyte-to-lymphocyte ratio (MLR);
- The platelet-to-lymphocyte-neutrophil fraction ratio (PLTLNFR);
- The systemic immune-inflammation index (SII);
- The systemic inflammation response index (SIRI); and
- The aggregate index of systemic inflammation (AISI).

The PLTLNFR was derived as an exploratory composite index combining platelet count and leukocyte differential components. Unlike the more widely used platelet-to-lymphocyte ratio (PLR), this index has not been extensively validated and should be interpreted as hypothesis-generating.

## Results

### Descriptive analysis

A total of 30 patients with neuroinvasive West Nile virus infection were included, of whom 17 (56.7%) presented with a predominantly neurologic phenotype (Septic = 0) and 13 (43.3%) with a septic phenotype (Septic = 1). Median age was 77 [66.5–81.0] years, with patients in the septic phenotype being slightly older than those in the neurologic phenotype (78 [72–84] vs. 73 [64–80] years), although this difference was not statistically significant. Overall, 16/30 (53.3%) patients were male, with a numerically higher proportion of males in the septic phenotype (61.5% vs. 47.1%), again without a significant between-group difference.

All patients were positive for both anti-WNV IgM and anti-WNV IgG antibodies (Table 1). Pre-existing heart failure was the most frequent comorbidity (19/30, 63.3%), followed by chronic obstructive pulmonary disease (COPD; 7/30, 23.3%) and active cancer (7/30, 23.3%). COPD was more common among patients with the septic phenotype (38.5% vs. 11.8%), whereas heart failure and active cancer were numerically more frequent in the neurologic phenotype; none of these differences reached statistical significance. Dialysis and baseline thrombocytopenia were uncommon (≤10% of cases) and similarly distributed between phenotypes.

At admission, the median white blood cell count was 7.75 × 10^9^/L [6.42–10.13]; neutrophils accounted for a median of 78.7% [77.0–81.5], and lymphocytes for 11.0% [8.0–13.6] of leukocytes. Platelet counts were modestly reduced, with a median of 179 × 10^9^/L [125.5–218.3], and thrombocytopenia was documented in 3/30 (10.0%) patients. Inflammatory indices showed values consistent with systemic inflammation in both phenotypes. These values were interpreted in the context of established adult reference ranges and previously published data in acute infectious and inflammatory conditions, rather than relative to a healthy control group. The median neutrophil-to-lymphocyte ratio (NLR) was 7.0 [5.31–10.75], the platelet-to-lymphocyte-neutrophil fraction ratio (PLTLNFR) 15.0 [9.1–20.1], and the systemic immune-inflammation index (SII) 1,211 [686–1,508]. Patients with the septic phenotype showed numerically higher NLR, SII, PLTLNFR and lower lymphocyte percentages compared with the neurologic phenotype, but these differences did not reach statistical significance (all *p* > 0.05; Table 1). C-reactive protein levels were elevated in both phenotypes, with no statistically significant difference between groups (Table 1).

All median values reported in the descriptive analysis refer to overall cohort distributions unless otherwise specified as phenotype-specific comparisons. Phenotype-specific differences are explicitly described when statistically significant or clinically relevant.

All hematological parameters were interpreted according to standard adult reference ranges routinely used at participating centers. Most median values, including MCH and leukocyte counts, remained within conventional reference limits despite statistically significant between-group differences.

Among all laboratory variables, only mean corpuscular hemoglobin (MCH) and monocyte percentage showed statistically significant between-phenotype differences at univariate comparison (Table 1), and were therefore further explored in regression modelling.

### Exploratory logistic regression analysis

Regression modelling was performed to explore whether selected routine laboratory parameters were associated with septic phenotype, given the exploratory aim and the observed between-group differences. The results of univariable and multivariable logistic regression analyses are summarized in Table 2. In univariable logistic regression models, lower mean corpuscular hemoglobin (MCH) and lower monocyte percentage were significantly associated with the septic phenotype. For MCH, the odds ratio (OR) per 1 g/dL increase was 0.37 (95% CI 0.17–0.79; *p* = 0.011), indicating that patients with lower MCH values were more likely to present with a septic phenotype. For monocytes, the OR per 1% increase was 0.73 (95% CI 0.54–1.00; *p* = 0.046). Other clinical variables and composite inflammatory indices (including the neutrophil-to-lymphocyte ratio (NLR), the systemic immune-inflammation index (SII), the systemic inflammation response index (SIRI) and PLTLNFR) showed only non-significant trends.

**Table 2:** Univariable and parsimonious multivariable logistic regression models for the association between MCH, monocyte percentage and the septic phenotype.

Variable	Univariable OR (95% CI)	Univariable *p* value	Multivariable *β* (SE)	Multivariable OR (95% CI)	Multivariable *p* value
Intercept (constant)			27.28 (12.57)		0.030
MCH, pg	0.37 (0.17–0.79)	0.011	−0.89 (0.43)	0.41 (0.18–0.95)	0.039
Monocytes, %	0.73 (0.54–1.00)	0.046	−0.33 (0.20)	0.72 (0.48–1.08)	0.110

MCH, mean corpuscular hemoglobin; *β*, regression coefficient; SE, standard error; OR, odds ratio; CI, confidence interval; MCH, mean corpuscular hemoglobin.

Given the limited number of events, a parsimonious multivariable model including only MCH and monocyte percentage was fitted in a complete-case subset (*n* = 25).

A multivariable logistic regression model was fitted with the septic phenotype (Septic = 1) as the dependent variable and mean corpuscular hemoglobin (MCH) and monocyte percentage as predictors, according to the equation logit[P(Septic)] = 27.28–0.89 × MCH − 0.33 × monocytes.

In this model, lower MCH remained independently associated with the septic phenotype (OR 0.41, 95% CI 0.18–0.95; *p* = 0.039), whereas monocyte percentage showed a non-significant trend (OR 0.72, 95% CI 0.48–1.08; *p* = 0.11).

Absolute monocyte count was also significantly associated with the septic phenotype in univariable analysis; due to collinearity with monocyte percentage and the limited number of events, it was not retained in the parsimonious multivariable model. The model showed good apparent discrimination (AUC 0.90); given the very small sample size, the low number of events, and the absence of internal or external validation, these performance estimates are likely substantially optimistic. The regression analyses were conducted exclusively for exploratory, hypothesis-generating purposes and should not be interpreted as evidence of predictive performance. Accordingly, these regression findings should be interpreted as hypothesis-generating signals rather than definitive predictors of phenotype.

## Discussion

In this retrospective multicenter exploratory study of 30 patients with neuroinvasive West Nile virus infection, we evaluated whether routinely available hematological and inflammatory parameters at admission were associated with distinct clinical phenotypes. In our cohort, classification of sepsis was based on Sepsis-3 criteria rather than microbiological confirmation alone; bacterial co-infection cannot be entirely excluded in this retrospective setting. Secondary bacterial infections during hospitalization have been reported in contemporary multicenter series of WNV and were associated with worse outcomes, supporting the need to consider infectious complications as confounders in retrospective cohorts (15). Nevertheless, subclinical or undetected co-infections cannot be entirely excluded in this retrospective analysis. Baseline demographic characteristics and major comorbidities were broadly comparable between the two groups, with no statistically significant differences in age, sex distribution, or underlying chronic conditions. These findings suggest that, in our series, the development of a septic presentation could not be readily attributed to differences in baseline frailty or comorbidity burden, nor could it be easily predicted by laboratory markers, in line with previous observations indicating that host response rather than baseline characteristics may drive clinical heterogeneity in WNV infection (11). Several hematologic parameters and composite inflammation-related indices were numerically elevated in both phenotypes, consistent with the presence of systemic inflammation and acute infection. Patients presenting with a septic phenotype exhibited numerically higher neutrophil-to-lymphocyte ratio (NLR), systemic immune-inflammation index (SII) and related composite indices, together with relatively lower lymphocyte percentages, suggesting a more pronounced inflammatory and possibly immune-dysregulated profile. Although these differences did not reach statistical significance, likely due to limited statistical power, the observed trends are biologically plausible and consistent with the known ability of WNV to induce lymphocytopenia and immune dysfunction (2, 16). Platelet-related parameters have been proposed as potentially informative in WNV as well; for example, platelet distribution width has recently been reported as a candidate prognostic/diagnostic marker in WNV infection (17). All patients were positive for both IgM and IgG antibodies, suggesting that most were evaluated during a subacute phase of infection rather than in the very early viremic stage. The timing of immune response maturation may have influenced the laboratory profile observed, and we cannot exclude that different biomarker patterns might emerge at earlier stages of infection.

In exploratory logistic regression analyses, lower mean corpuscular hemoglobin (MCH) and lower monocyte percentage were associated with the septic phenotype in univariable models, and MCH remained independently associated with sepsis in a parsimonious multivariable model including only MCH and monocytes. These findings raise the hypothesis that subtle alterations in erythrocyte indices and monocyte counts may reflect a more severe systemic inflammatory response, bone marrow dysfunction or inflammation-related anemia in patients who develop sepsis in the context of neuroinvasive WNV infection. While specific data on erythrocyte indices in WNV infection are scarce, similar associations between red blood cell parameters, inflammation and adverse outcomes have been reported in bacterial sepsis and other severe infectious (18, 19). It should be emphasized that the regression models were not developed as clinical prediction tools but as exploratory analyses intended to identify potential signals worthy of further investigation. The limited events-per-variable ratio and the absence of model validation preclude any inference regarding clinical applicability.

Importantly, although the between-group difference in MCH reached statistical significance, the absolute median difference between phenotypes was approximately 2 pg. and remained within conventional reference ranges. This distinction highlights the difference between statistical significance and clinical relevance. From a practical perspective, such a small variation in MCH would not independently alter clinical decision-making and may reflect subtle inflammation-related hematologic modulation rather than a clinically actionable biomarker. Therefore, the observed association should be interpreted cautiously as hypothesis-generating rather than clinically directive.

The paucity of published data on detailed laboratory phenotyping in neuroinvasive WNV infection represents an important knowledge gap. Most available studies focus on clinical presentation, neuroimaging findings and long-term neurological sequelae, rather than on routine hematologic and inflammatory biomarkers (8, 20). Nonetheless, recent work has increasingly emphasized the role of host inflammatory responses in WNND, including peripheral biomarkers of neuroinflammation and cytokine signatures potentially associated with disease severity. A recent systematic review summarized the heterogeneity of inflammatory patterns reported in WNND and underscored the need for clinically accessible markers, while peripheral biomarker studies have explored serum inflammatory and neuronal injury signals in neuroinvasive infection (21, 22). In contrast, a growing body of literature in sepsis and severe viral infections has demonstrated that indices such as NLR, SII and related composite markers are associated with disease severity, organ dysfunction and mortality (19, 23). Our findings, characterized by elevated inflammatory indices in both phenotypes and numerically higher values in septic cases, are broadly consistent with this evidence, even though we were unable to demonstrate statistically significant between-group differences. Multinational observational data have also highlighted the clinical heterogeneity and prognostic stratification challenges in WNND, supporting the need for improved risk models integrating clinical and laboratory variables (24).

Furthermore, we identified a potential role for two laboratory indices, namely the neutrophil-to-lymphocyte ratio (NLR), as reported for other viral infections, and mean corpuscular hemoglobin (MCH), that may help distinguish patients with neurological manifestations from those with septic presentations. The clinical utility of these findings for guiding disease management should be evaluated in larger cohorts. Other limitations of our study include its retrospective design and the very small sample size, which substantially limit statistical power and increase the risk of both type I and type II errors. Moreover, although deliberately restricted to two predictors, the multivariable logistic regression model remains at risk of overfitting and optimistic performance estimates and lacks external validation. Residual confounding due to unmeasured clinical factors, variability in the timing of blood sampling, and differences in supportive care across centers cannot be excluded (25). Moreover, persistent WNV IgM responses have been described, which may complicate strict interpretation of serology in relation to infection timing.” (26).

Although none of the between-phenotype differences reached statistical significance in this small cohort, the consistent numerical trends observed in inflammatory indices suggest that clinically relevant differences may emerge with larger sample sizes. Neuroinvasive West Nile virus infection is a rare condition, and adequately powered analyses will likely require coordinated, multi-center efforts.

Given the retrospective design, the small sample size, and the absence of external validation, these associations should be considered hypothesis-generating and require confirmation in larger, prospectively characterized multicenter cohorts before any clinical implementation.

## Conclusion

In conclusion, in this small exploratory cohort of patients with neuroinvasive West Nile virus infection, subtle differences in selected routine hematological parameters were statistically associated with a septic clinical presentation. Given the retrospective design, limited sample size, and lack of validation, these findings should be considered hypothesis-generating. Larger, prospectively characterized multicenter studies are required to determine whether such laboratory patterns have reproducible diagnostic or prognostic significance.

## Data Availability

The raw data supporting the conclusions of this article will be made available by the authors, without undue reservation.
